# The Influence of Pension Mode on the Mental Health of Older Adults—Evidence from Older Adults in China

**DOI:** 10.3390/ijerph19010119

**Published:** 2021-12-23

**Authors:** Liqing Li, Luyao Yu

**Affiliations:** School of Public Management and Law, Hunan Agriculture University, Changsha 410125, China; liliqing1136@163.com

**Keywords:** successful aging, old-age care methods, mental health, older adult

## Abstract

Successful aging is achieved throughout the life course, and successful aging groups tend to have good psychosocial and physical conditions and are active in social activities. With increasing age, the mental health problems of older adults have become increasingly prominent, and the choice of pension mode is closely related to the mental health of older adults. Starting from the psychological level of the older adult, this paper used data from the 2018 Chinese Longitudinal Healthy Longevity Survey to study the impact of three pension methods on the mental health of older adults. The study found that, at present, there are three types of pension modes in China: living alone, family pension, and institutional care, and family pensions are still the mainstream pension mode. Older adults with deeper negative feelings are more inclined to family pensions than to live alone, but the spiritual comfort provided by family members does not improve the negative feelings of older adults. Institutional care deepens the negative feeling and reduces the positive feeling of older adults. In addition, retirement or pension and medical insurance, as life security in old age, can effectively reduce the negative feelings of old age and promote positive feelings. In view of the present situation of China’s pension mode and the psychological characteristics of the older adults, we should further build a perfect family pension security system, promote the personalized service construction of older adult care institutions, promote applicable aging renovation of existing residential areas, and encourage older adults to engage in healthy exercise.

## 1. Introduction

Data from the 7th China’s National Census in 2021 show that there are currently 260 million people over the age of 60 in China, accounting for 18.7% of the country’s total population, and in 2020, the dependency ratio of China’s aging population rose to 16.9%. The scale of aging is unprecedented and an increased number of psychological problems have occurred because of the rising number of persons surviving into old age. After entering old age, due to changes in the health status and social functions of the body, the worldview of older adults has changed, and these changes have caused a certain gap in the psychology of the older adult, thus causing a series of changes in psychological conditions. According to a survey conducted by the National Health Commission, 30.3% of older adults in urban areas have a mental health problem, while only 26.8% of older adults in rural areas do. Mental health problems in older adults directly affect their participation in social activities and physical health level, and the breakdown of family and social relationships caused by psychological problems severely affect the quality of life of older adults and the harmony and stability of the entire society. Therefore, much attention has been given to how to solve the mental health problems of the older adult population, improve the quality of life for the older adult population, and promote a harmonious society.

With advanced age, the mental health of older people in different situational stages is influenced and restricted by many factors, such as education level, economic income, physical condition, and family structure. In addition, the pension mode is also one of the main factors. As economic development and social changes continue to accelerate, the traditional family structure begins to shift [1]. At the same time, the One-Child Policy dramatically changed the Chinese family structure [2]. While intergenerational family support still plays an important function in the current senior care system, there is also an increasing number of older adults living alone and in institutions. How to achieve “older adult care”, “happiness”, and “old-age security” is an urgent problem to be solved.

Therefore, this study explores the impact of older adult care on mental health, focusing on the psychological problems of older adults in family care, living alone, and in institutional care, to improve the quality of life of older adults and achieve successful and active aging.

## 2. Literature Review and Analysis Framework

### 2.1. Research on the Definition of Mental Health

Mental health is an ongoing and actively developing state of well-being. In 2001, the World Health Organization proposed that mental health is a state of health or well-being in which individuals can realize themselves, cope positively with the stresses of daily life, work productively, and have the ability to contribute to the society in which they live. The psychologist Maslow believed that mental health is not only the health of the “inner and secret” psychological world of people, and research on mental health should be directed to the field of people’s lives [3]. Other scholars have pointed out that mental health is a good adaptive state in which individuals are in self-harmony and interact positively with the environment and can give full play to their psychological potential [4]. As older adults enter their later years, their mental health also presents different characteristics, with changes in their social roles and environment [5]. Liu Xiao qin defined the mental health of older adults in five aspects: normal cognitive function, positive and stable mood, appropriate self-evaluation, harmonious interpersonal communication, and good adaptability [6].

### 2.2. Successful Aging

Mental health problems of older adults are closely related to the formulation and implementation of policies around successful ageing and the Health China Strategy [7]. Successful aging is a new concept put forward in the context of population aging. The earliest definition of successful aging is “the most satisfying and happy life state achieved by individuals” [8]. As research continues, the meaning of successful aging is being refined. It was not until 1987 that Rowe J W and Kahn R L pioneered the concepts of normal aging and successful aging [9]. The importance of successful aging has been emphasized in recent years, with the focus shifting towards attaining healthier aging rather than longevity [10]. Scholars differ on what constitutes as successful ageing. Pachana NA showed that factors affecting successful aging include social activities and psychological and physiological health factors, and the differences in these factors lead to different views on successful aging [11]. Geard pointed out in his latest study that successful aging can be defined as a late-life process characterized by changes in height and psychological, cognitive, and social functioning [12]. From the perspective of individual aging, Mu Guangzong proposed the concept of “old age loss” and “old age gain”. If the positive energy of “old age gain” can balance the negative energy of “old age loss”, the development of aging is a positive trend, i.e., successful aging [13]. Some scholars have conducted empirical studies. Yang Ni scholars analyzed successful aging from the perspective of “old drifters” based on three dimensions: economic, social integration, and health dimensions [14]. Other scholars, Zimmer. C and Nia Chen, focused on the impact of social participation and successful aging [15,16].

### 2.3. The Influence of Pension Mode on the Mental Health of the Older Adult

A positive aging attitude is an important sign for older adults to achieve successful aging [17]. Meanwhile, improving and enhancing the mental health of older adults is essential to achieve the goals of healthy aging and active aging in China [18]. Existing literature has studied the influential factors of the mental health of older adults from the perspectives of individual characteristics [19,20,21], social cognition [22,23,24], environmental characteristics [25,26,27], and so on.

Different pension modes are manifestations of the interaction of social and environmental factors, and the selection of appropriate pension modes has an important impact on the quality of life of older adults in their later years [28]. From another perspective, this paper studies the influence of the pension mode on the mental health of older adults. Chen Dong studied the influence of three pension modes, namely, family pension, social pension, and self-pension, on the happiness of the rural older adult in various dimensions. The results show that among the three pension modes, the self-pension mode has the highest impact on older adults’ degree of happiness, but the family pension still plays an important role. Meanwhile, a comparison between empty nesters and nonempty nesters shows that the happiness of empty nesters is more dependent on self-economic ability and service facilities for the older adult [29]. Cheng, Joyce M noted in his study that homebound older adults experienced both physical and mental health challenges that restricted their ability to participate in activities of daily living, recreation, and social interactions. Thus, we should take measures to address their isolation and loneliness [30]. Research by Shen Ke and others indicates that the multigeneration living model can effectively alleviate the depression tendency of older adults and improve their life satisfaction. Special emphasis was placed on the fact that the presence of children significantly improved the well-being of widowed, divorced, or unmarried older adults [31]. Zunzunegui M V also has a similar view. Through their research in Spain, they concluded that emotional support from children plays an important role in maintaining the physical and mental health of older adults [32]. Xu’s research also shows that living with children has a positive effect on the mental health of older adults, but this effect depends both on the gender of the children and the space in the shared dwelling of the children living with the older adult. In contrast, living with a daughter was most beneficial to the mental health of older adults [33]. While Kharicha’s study also indicates that living with children improves mental health in older adults, the findings also suggest that living alone is not necessarily bad for health [34]. Scholars have also proposed a different view. Hughe et al. found a potential association between family structure and self-rated health, mobility, and depression tendencies in older adults. Their survey showed that older adults who lived with their children had more depressive symptoms than those who only lived with their spouses [35]. Morioka also suggested that because of the difference in lifestyle and habits between the two generations, living with the older adult and their children easily produces intergenerational contradictions, which have a negative impact on the physical and mental health of older adults [36]. Merz et al. found that intergenerational support for receiving instrumental help from children was negatively associated with subjective well-being among older adults and that the quality of intergenerational relationships was the most significant predictor of subjective well-being. [37].

### 2.4. Analysis Framework

**Figure 1:**
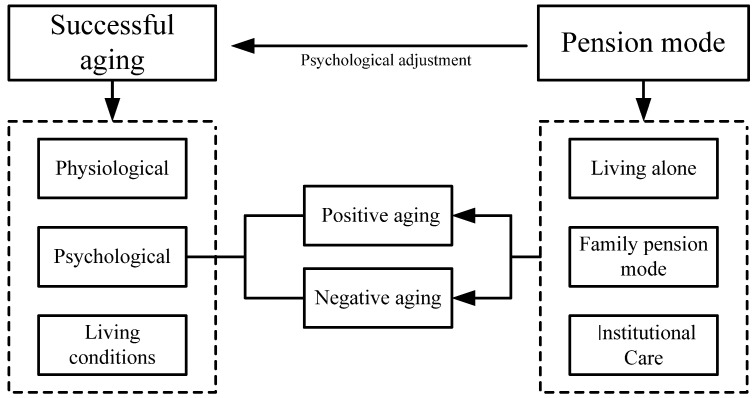
Analysis framework of the influence of pension mode on the mental health of the older adult.

Many scholars have conducted studies on the factors that affect the mental health of older adults. but more often considers the effects of living alone versus not living alone and the emotional support of children for older adults. At the same time, the evaluation of the mental health of older adults is mostly based on depression tendency, and the measurement index of mental health is relatively single. The mental health of older adults needs to be measured from many aspects. In view of this, based on the national survey data, this paper will analyze the living conditions of older adults in China under different pension modes, and discuss the impact of pension modes on mental health. It is unclear to what extent the choice of pension modes will have an impact on the mental health of the elderly, and what the impact of different pension modes are on older adults. Therefore, the research on the influence of pension mode on the mental health of older adults is helpful to grasp the psychological characteristics of older adults to improve their mental health, so as to improve the quality of life to achieve successful aging. The analysis framework of this paper is constructed based on the above content, as shown in Figure 1.

## 3. Data Description and Model Setting

### 3.1. Data Source

The data for this article were obtained from the 2018 Chinese Longitudinal Healthy Longevity Survey, covering 23 of 31 provinces, cities, or autonomous regions in China. Questionnaires were administered to older adults aged 65 years and older and adult children aged 35–64 years. The respondents’ questionnaires covered the following topics: basic older adult and family conditions, socioeconomic background and family structure, economic sources and economic status, self-assessment of health and quality of life, cognitive function, personality and psychological characteristics, ability to perform daily activities, lifestyle, life care, illness treatment, and medical cost bearing. The original sample size was 15,874 groups, and according to the purpose and need of this study, after screening and eliminating the samples with serious missing cases, the final study data were 5930 groups.

### 3.2. Variable Setting

The independent variable (IV1) is the old-age pension method of older adults. Since there is no direct counterpart in the questionnaire, we chose the question “Who do you live with now?” [38]. The answers are family members (including nannies), living alone, and pension institutions. This paper takes family pensions as the reference group and establishes the dummy variables “living alone” and “institutional care”.

The dependent variable (DV) is the mental health status of older adults. Mental health scales are numerous and more complex to measure [39]. Through a literature review, we found that the domestic and international mental health conditions of older adults have been studied using many assessment tools and scales. Using the existing research on mental health indicators [40], a system of psychological indicators of successful aging has been constructed. This paper divides the mental health of the aged positive and negative individuals. Positive aging primarily represents positive mental health in older adults, while negative aging represents the negative mental health of older adults; see Table 1 for details. The geriatric negative index and the positive index were derived from the total score of the three questions. The higher the positive score in old age, the lower the positive feeling in old age, and similarly, the higher the score, the lower the negative feeling in old age. Cronbach’s alpha coefficient was further used to evaluate the reliability of the constructed index system. The internal consistency reliability coefficients of the mental health indicator system are 0.833 and 0.799, which indicates that the indicator system is effective.

According to the existing research results [41,42] and based on the concept of successful aging, this paper selects control variables (CV) from DC (demographic characteristics), HL (health level), FC (family characteristics), and lifestyle. Demographic characteristics include the older adult’s household registration (urban and rural), gender, age, MS (marital status), EDUL (education level), PRB (pensions and retirement benefits), and MI (medical insurance). The HL (health level) of the older adult is mainly composed of two observation variables: SAH (self-assessment of health) and DAA (daily activity ability). Self-rated health is the subject’s subjective feeling about their own health, and self-rated health can reflect both subjective and objective aspects of health status. Daily activity ability tests cover a wide range of areas and comprehensively evaluate the physical health of older adult individuals. The tests consist of six questions in total, and the higher the score, the more serious the impairment of the daily activity ability. FC (family characteristics) are potential exogenous variables, which are mainly composed of two observational variables: FLS (family living standard) and FPEC (family provides emotional comfort). LS (lifestyle) was a potential exogenous variable, which was composed of two observational variables, SF (socialization with friends) and behavioral habits of the older adult; see Table 2 for details.

To eliminate the possible endogeneity in the older adult’s aging style, two instrumental variables (IV2), “number of children” and “self-assessment of life status”, were selected. The reason for choosing the number of children is the deep-rooted belief in the older generation that “many children make a good family” and “raising children for old age”. Similarly, it is generally the case that the larger the number of children, the more likely they are to share the responsibility of care for the older adult, either by choosing one child to take care of them or by living alone and having children take care of them in turn. Conversely, seniors without children are more likely to choose pension institutions. The choice of self-assessment of living status is based on older adults’ satisfaction with life. Relatively speaking, older adults who are dissatisfied with their current life situation, as their children choose to go out to work to make a living, further affect the possibility of living alone and Institutional Care.

### 3.3. Descriptive Statistics

The mean value and standard difference of each variable are calculated. The descriptive statistics of the three categories of variables are shown in Table 3. In general, the mental health of the older adult is relatively positive. From the perspective of the choice of pension mode, the older adult who chooses a family pension has the highest positive feelings of old age, while the older adult who lives alone has more serious negative feelings of old age. The analysis shows that this generation of older adult people has more traditional thoughts. Influenced by the idea of “raising children for old age”, 82.45% of the older adult choose family (including nannies) for pensions, while only 2.97% choose institutions for pensions. Family pensions are still the mainstream of the older adult population in China. At the same time, it also reflects that the construction of China’s social pension service system is in its infancy, and the supply of pension services is insufficient.

Among the variables reflecting geriatric demographic characteristics, males accounted for 45% of the overall sample, and 48% of male older adults chose to age with their families. The average age of older adults in the sample was 83.35. Urban older adults accounted for only 31% of the overall older adults, with urban older adults more likely to choose older adult care institutions and rural older adults more likely to choose to live alone. Unmarried older adults were more likely to choose to live alone and in older adult care institutions. The average number of years of education received by older adult is only 3 years, and the overall education level is low. Older adults with pensions and medical insurance more often choose to live in institutions; older adults living alone with medical insurance account for 90% of all the older adults living alone. Among the variables that reflect the health level of the older adult, most of the older adults think their health is good. The average value of the older adult with impaired daily activities is 6.74, and the older adult with more serious impairment in daily activities will choose more institutional care and family pensions. Among the variables reflecting the family characteristics of older adults, the older adult with a good living standard would more often choose family retirement and living alone. Older adults who chose family retirement can enjoy the spiritual comfort provided by more loved ones. Among the variables reflecting the lifestyle characteristics of older adults, those who live alone have more frequent visits to their homes and friends than the other two modes of ageing; the number of older adults who smoke in nursing homes is the lowest. In terms of instrumental variables, the mean value of older adults’ children in the sample was 3.93, and the mean value of self-assessment of life status was 2.06.

### 3.4. Model Setting

In the study of physical and mental health of older adults, three models are more commonly used: multiple linear regression, ordered probit regression, and ordered logit regression. The multiple linear regression model is one of the most widely used models, and it has the advantage that the model is simple and straightforward, easy to understand, and widely applied, and can be used to study a variety of issues. However, for the study of mental health, it exists conceptually, but its precise value is difficult to obtain in principle and practice. Ordered probit regression models transform mental health variables that are originally continuous but cannot be measured precisely into discrete variables that can be measured, and when the dependent variable cannot be described exactly, ordered probit regression models can classify the dependent variable and essentially explain the relationship between mental health and various influencing factors. The object of this paper is the mental health of the elderly, which as a subjective evaluation index is affected by demographic variables, sociological variables, and subjective factors, and at the same time to study the influence of different aging methods on the mental health of the older adult, Based on the obtained data an ordered probit should be taken for estimation. The model is set as follows:

(1) $$MH_{i}=a_{0}+a_{1}PM_{i}+a_{2}X_{i}+\varepsilon_{i}$$

where i represents different individuals, $MH_{i}$ is the mental health of the older adult, $PM_{i}$ represents the pension mode, X is the control variable, $\varepsilon_{i}$ is the random disturbance term, *a*_0_ is the intercept term, *a*_1_ is the coefficient of the independent variable, and *a*_2_ is the coefficient of the control variable.

On the basis of the ordered probit model, this paper established an OLS model to test the stability of the model:

(2) $$MH_{i}=b_{0}+b_{1}PM_{i}+b_{2}X_{i}+\varepsilon_{i}$$

where $MH_{i}$ has the same meaning as in Equation (1), $b_{0}$, $b_{1}$, and $b_{2}$ are regression coefficients, $\varepsilon_{i}$ is the random error term, and the other variables have the same meaning as in Equation (1).

As mentioned earlier, there may be endogeneity in the choice of pension mode. The reason may be due to omitted errors in the variables or a bidirectional causal relationship between the pension mode and the mental health of older adult. In this paper, we adopt the two-stage least squares (2SLS) method based on one-stage regression to eliminate the endogeneity of the model using instrumental variable method. An effective instrumental variable needs to satisfy both instrumental variable correlation and instrumental variable exogeneity, i.e., correlated with the endogenous variables, but at the same time uncorrelated with the residual terms. Combining the previous research results with the model characteristics of this paper, the instrumental variables selected in this paper are the number of children of an older adult and the self-assessment of life status.

Based on the previous OLS model, the econometric model of 2SLS instrumental variable method is designed as follows:

(3) $$PM_{i}=\gamma_{0}+\gamma_{1}IV_{1}+\gamma_{2}IV_{2}+\delta X_{I}+\varepsilon_{i}$$

(4) $$MH_{i}=b_{0}+b_{1}PM_{i}+b_{2}X_{i}+\varepsilon_{i}$$

Among them, Equation (3) is the 2SLS first-stage regression, where $IV_{1}$ $IV_{2}$ represent the two instrumental variables, and $\gamma_{1}$ $\gamma_{2}$ are the instrumental variable coefficients, which can be judged by their F statistics whether they are weak instrumental variables. Equation (4) is the 2SLS second-stage regression, and the other coefficients are estimated by the dependent variable PM derived from the first-stage regression.

## 4. Empirical Analysis and Results

### 4.1. Positive Aging: The Effect of Pension Mode on the Positive Mental Health of the Older Adult

Table 4 shows the results of the regression using the ordered probit model, where Model (I) demonstrates the effect of the independent variables on active old age. The regression results showed that compared with living alone, a family pension significantly improved the positive feelings of the older adult; that is, older adults who chose a family pension had more positive psychological health, and nursing home living had reduced geriatric positivity, but not significantly.

In the demographic characteristic variables, the increase in age significantly reduced the positive feeling of older adults, and the older the positive feeling of old age was, the lower the positive mental health score was. Pension(s) significantly affected positivity in old age, possibly because they act as security in old age, giving older people the opportunity to engage in more meaningful activities instead of fighting for a living. At the same time, older adults with medical insurance possess a higher sense of aging positivity, and a higher reimbursement ratio of medical insurance can give older adults a sense of security, thus improving the mental health level of older adults. In the variables of health level, self-rated health has a significant positive impact on the positive feeling of old age; that is, older people who think they are healthier have a higher positive feeling of old age. Older adults with more impaired daily activities reported lower levels of positivity in old age. Among the family characteristic variables, economic condition has a significant impact on older adults’ positive feelings, and older adults with better living conditions have better mental health. In terms of lifestyle, older adults who regularly exercise have a higher sense of positivity in old age, and good physical quality is the premise of mental health. It is noteworthy that smoking and drinking can significantly improve older adult positivity, possibly because older adult people tend to use smoking and drinking to relieve their psychological emotions.

### 4.2. Negative Aging: The Negative Impact of Pension Mode on the Mental Health of Older Adults

Model (II) in Table 4 shows the regression results of the impact of independent variables on the negative mental health of older adults. It was be found that older adults who choose a family pension have significantly reduced negative feelings, and the negative psychological score is lower. The conclusion of pension institutions is consistent, but not significant, which may be due to the endogeneity of pension methods.

Among the demographic characteristic variables, compared with the older adult without a spouse, older adults with a spouse have lower negative feelings of old age, which indicates that their spouse can effectively provide psychological guidance to reduce the negative emotions of older adults. In the health level variable, older people with better self-rated health will have a lower negative feeling in old age, which is consistent with the previous conclusion. The severity of impaired ability of daily activities has a significant effect on the negative feelings of older adults, which indicates that older adults with impaired ability of daily activities are more prone to negative psychology, thus aggravating the negative feelings of the older adult. Regarding the family characteristic variables, the negative feeling of older adult individuals with lower living standards was greater than that of older adult individuals with better living standards. Family spiritual comfort can effectively relieve the negative emotions of older adults and reduce the negative feelings of older adults. In terms of lifestyle, smoking and drinking can significantly reduce the negative feeling of old age, which is consistent with the previous conclusion. Meanwhile, physical exercise can reduce the negative feeling of old age, which indicates that older adult individuals who engage in regular physical exercise have a better mentality and better ability to eliminate worries.

### 4.3. Robustness Test of the Model

To estimate the robustness of the results, Table 5 shows the regression results of the OLS model for model (III) and model (IV), comparing them with the results of model (I) and model (II). The results show that the OLS and ordered probit models have not been the regression results in the influence symbol with significant contradiction to appear on the results. Although there are differences in coefficients due to different methods, it can be considered that the regression results are robust.

### 4.4. Elimination of Endogeneity

This paper aims to explore the net effect of pension mode on the mental health of older adults. However, due to the existence of endogeneity problems, the estimation results may be inaccurate. The two-way causal relationship between pension mode and mental health of the older adult is the main endogenous cause; that is, the pension mode affects the mental health of older adults. However, the mental health level of older adults will also have a certain impact on the choice of pension method. To further verify the robustness of the results, a 2SLS model was established on the basis of the OLS model.

The Hausman test of the model showed that positive and negative ageing was significant at the 1% level of significance, which proves the endogeneity of the OLS model compared to the instrumental variable 2SLS model; therefore, the two-stage 2SLS regression analysis using instrumental variable IV is valid. At the same time, a weak instrumental variable was tested for instrumental variable IV, and the results showed that when A and B were larger than the critical value of 10% error in C, the null hypothesis could be rejected, and no weak instrumental variable problem could be considered. Table 6 shows the regression results of the 2SLS model established by instrumental variables, which are compared with the original OLS model. Models (V) and (VI) represent the results obtained after the “number of children” and “self-assessment of life status” are used as instrumental variables to establish the 2SLS model to eliminate the endogeneity of pension mode selection.

In terms of positive aging, the significance of nursing homes for positive aging increased after eliminating endogeneity, indicating low levels of positive psychological well-being among older adults living in nursing homes. Among the personal characteristic variables, the effect of household registration on the positive feeling of old age changed from positive to negative with increased significance, indicating that the positive feeling of urban older adults is greater than that of rural older adults. This is attributed to the fact that the living standard conditions of urban older adults are better than those of rural older adults, and material satisfaction reduces the psychological burden. The effect of gender on active old age changed from positive to negative but was not significant. Marital status, after accounting for endogeneity, resulted in lower positive aging among older adults with spouses. The significance of daily activity ability disappeared; economic level no longer had a significant positive influence on the older adult, and the influence turned from positive to negative. The effect of family providing spiritual consolation on the positive feelings of the older adult changed from negative to positive and increased significantly, which may indicate that older adults have not been fully understood and respected by the family due to the generation gap in emotional communication with family members and failed to solve their worries in a timely manner. The influence of visiting and socializing with friends on senile positivity changed from positive to negative. The effect of smoking and drinking on the positive feeling of old age disappeared, and the effect of smoking changed from negative to positive.

In terms of negative aging, after taking into account endogenous effects, the negative feeling of older adults who choose a family pension is still less than that of living alone. Institutional endowment can reduce the positive sense of old age and enhance the negative sense of old age. Among the demographic characteristic variables, the negative feeling of the older adult in urban areas is less than that in rural areas after considering the effect of endogeneity. The negative feelings of old age increased significantly with age, and the negative feelings of old age deepened gradually with age. The significance of years of education on the negative feeling of old age disappeared. Older adults with pensions and medical insurance have lower negative feelings of old age, but the significant effect of economic level on negative feelings of old age disappears. The negative effect of visiting and socializing with friends on old age disappeared significantly.

## 5. Conclusions

First, currently, family pension is still the mainstream. Family pensions significantly improve positive feelings of older adults and reduce negative feelings. However, it is worth noting that although the family provide spiritual consolation to older adults, it is still not a good mental state. Living with family, subject to the differences between two generations, leads to friction. In addition, the older adult may need to take on more responsibility in the family, facing more stress.

Second, from an urban-rural perspective, after controlling for endogeneity, urban older adults have a higher sense of positive ageing than those in rural areas, meaning that urban older adults are more psychologically optimistic. Compared with older adults in urban areas, rural older adults living alone are more common, and the disparity due to the difference in economic income can make them more likely to fall into negative feelings. Returning to an essential issue, the development model of the urban-rural dichotomy makes unbalanced and inadequate development between urban and rural areas gradually revealed in the field of older adult care. Based on such a realistic background, we should pay more attention to the realization of successful aging of older adults in rural areas. Compared with living alone, the negative feelings of old age were significantly reduced by institutional care. However, when endogeneity was considered, the negative feelings of old age were changed from positive to negative by institutional care, indicating that there was endogeneity in older adult people choosing institutional care.

Third, the effect of pension mode on the mental health of older adults varies with personal characteristics, health level, family characteristics, and lifestyle. Meanwhile, geriatric positivity decreased with increasing age. Older adults living with a spouse did not improve their positive feelings of old age but deepened their negative feelings. Pension and medical insurance, as life security in old age, can effectively reduce negativity in old age and promote positive feelings.

## 6. Discussion

Through the establishment of the psychological index system of the older adult population, this paper analyzes the influence of pension methods on the mental health of older adults. Therefore, the following countermeasures and suggestions are proposed for older adult groups with three pension methods.

First, a sound family old-age security system should be built. The changes in family structure brought about by the family planning policy have weakened the function of traditional families to provide for the aged. At the same time, the problem of providing for the aged in urban and rural areas has gradually emerged. For older adults in rural areas, the government should increase rural pension subsidies and include the development of pension services and facilities in the rural revitalization plan. As the first choice for the majority of older adults, the government should implement the long-term care insurance system, raise relevant family subsidies, and improve the “third bed” of old-age services. At the same time, it promotes the development of smart old-age services and forms a new old-age model featuring family participation in the early stage and social service in the later stage. While giving full play to the traditional family aging function, children and family members should better perform the role of the “listener”, learn to put themselves in others’ shoes, and provide timely psychological counselling. Older couples should be psychologically compatible, and respect, love, trust, and understand each other.

Second, a diversified construction of older adult care institutions should be promoted. Older adults living in institutions are in a disadvantageous position in terms of daily activities and mental health, so diversified pension service institutions should meet the personalized needs of older adults, pay attention to the emotional communication of older adults, encourage them to carry out positive and reasonable social activities, avoid cognitive degradation and psychological problems, and pay more attention to the mental and physical health of the older adult group with dementia and disability.

Third, applicable aging renovation of existing residential areas should be promoted and older adults should be encouraged to engage in healthy exercise. On the one hand, the government and the community need to pay more attention to older adults living alone and care about their living condition, physical condition, and mental state. improve their sense of residential security and promote the community embedded pension mode. On the other hand, physical health is closely related to mental health, and older people who regularly participate in physical exercise tend to be in good mental condition, so they should be encouraged to engage in intellectual and physical activities, to keep older adults mentally active [43].

## Figures and Tables

**Table 1:** Mental health index system of the older adult population.

Index Name	Indicator Name	Score
Positive mental health (Positive aging)	Positive attitude towards life	1–5
	Energetic	
	Hale and hearty	
Negative mental health (Negative aging)	Feelings of loneliness	1–5
	Feel useless	
	Sad or depressed	

**Table 2:** Introduction of related variables.

	Variable Name	Variable Code
DV	Positive aging	Continuous variable
	Negative aging	Continuous variable
IV (1)	Pension mode	Family pension: 1 = Family; 0 = Living alone Institution care: 1 = Institution; 0 = Living alone
CV	hukou	0 = rural residents; 1 = urban residents
	gender	0 = Female; 1 = Male
	age	Continuous variable
	MS	0 = Unmarried (including widowed and divorced; 1 = Married
	EDUL	Continuous variable
	PRB	0 = NO; 1 = Yes
	MI	0 = NO; 1 = Yes
	SAH	1 = very good; 2 = good; 3 = In general; 4 = not good; 5 = very bad
	DAA	1 = No help needed; 2 = A little help needed; 3 = Full help needed
	FLS	1 = very rich; 2 = relatively rich; 3 = average; 4 = relatively difficult; 5 = very difficult
	FPEC	0 = negative; 1 = positive
	SF	0 = negative; 1 = positive
	Smoking	0 = negative; 1 = positive
	Drinking	0 = negative; 1 = positive
	Exercise	0 = negative; 1 = positive
IV (2)	Number of children	0–13
	self-assessment of life status	1 = very good; 2 = good; 3 = In general; 4 = not good; 5 = very bad

**Table 3:** Descriptive statistics of variables.

Variable Type	Variable	All Samples		Family Pension		Living Alone		Institution Care	
		AVG	STD	AVG	STD	AVG	STD	AVG	STD
IV (1)	Positive aging	7.36	2.25	7.31	2.25	7.57	2.22	7.71	2.14
	Negative aging	11.56	2.34	11.67	2.18	11.02	2.41	11.16	2.37
DV	Pension mode								
	FP: 0 = LA	0.15	0.35						
	IC: 0 = LA	0.03	0.18						
CV	DC								
	hukou	0.31	0.46	0.30	0.46	0.25	0.43	0.72	0.45
	gender	0.45	0.50	0.48	0.50	0.34	0.48	0.39	0.49
	age	83.35	11.49	82.88	11.80	84.92	9.63	88.52	8.87
	MS	0.50	0.50	0.58	0.49	0.10	0.30	0.16	0.37
	EDUL	3.81	4.53	3.96	4.58	2.73	3.95	4.77	5.11
	PRB	0.51	0.50	0.51	0.50	0.48	0.50	0.73	0.45
	MI	0.89	0.31	0.89	0.31	0.90	0.30	0.77	0.42
	HL								
	SAH	2.52	0.89	2,52	0.89	2.50	0.90	2.56	0.89
	DAA	6.74	1.97	6.77	1.99	6.35	1.30	8.11	2.98
	FC								
	FLS	2.85	0.62	2.84	0.62	2.93	0.62	2.76	0.57
	FPEC	0.99	0.07	1.00	0.05	0.99	0.11	0.93	0.25
	LS								
	SF	0.63	0.48	0.62	0.48	0.69	0.46	0.44	0.50
	Smoking	0.15	0.36	0.16	0.37	0.13	0.34	0.08	0.27
	Drinking	0.15	0.36	0.15	0.36	0.14	0.35	0.15	0.36
	Exercise	0.34	0.47	0.34	0.47	0.33	0.47	0.41	0.49
IV (2)	Number of children	3.93	1.96	3.87	1.96	4.35	1.92	3.46	1.78
	Self-assessment of life status	2.06	0.80	2.04	0.79	2.18	0.79	2.16	0.80

**Table 4:** Regression results of the ordered probit model of factors affecting the mental health of the older adult.

	Model (I)	Model (III)	Model (II)	Model (IV)
FP: 0 = LA	−0.145 *** (0.041)	−0.275 *** (0.077)	0.220 *** (0.041)	0.459 *** (0.081)
DC				
age	0.004 *** (0.002)	0.008 *** (0.003)		
MS			0.160 *** (0.036)	0.315 *** (0.071)
EDUL			0.014 *** (0.004)	0.027 *** (0.007)
PRB	−0.086 *** (0.030)	−0.165 *** (0.055)		
MI	−0.136 *** (0.043)	−0.245 *** (0.080)		
HL				
SAH	0.592 *** (0.016)	1.101 *** (0.029)	−0.442 *** (0.016)	−0.087 *** (0.031)
DAA	0.034 *** (0.008)	0.064 *** (0.014)	−0.020 *** (0.008)	−0.044 *** (0.015)
FC				
FLS	0.179 *** (0.022)	0.332 *** (0.042)	−0.162 *** (0.023)	−0.321 *** (0.044)
FPEC			0.372 ** (0.181)	0.810 ** (0.354)
LS				
SF			−0.077 ** (0.031)	−0.131 ** (0.060)
smoking	−0.085 ** (0.040)	−0.160 ** (0.076)	0.097 ** (0.041)	0.197 ** (0.080)
drinking	−0.092 ** (0.040)	−0.172 ** (0.074)	0.131 *** (0.040)	0.243 *** (0.078)
exercise	−0.361 *** (0.030)	−0.685 *** (0.056)	0.216 *** (0.030)	0.410 *** (0.059)
_cons		2.839 (0.459)		13.376 (0.483)
Pseudo R2	0.077		0.052	
Ajd.R2		0.283		0.198

Note: ***, **, represent significance levels of 1%, 5%, respectively, and standard error terms are in parentheses.

**Table 5:** OLS model regression results.

	Model (I)	Model (III)	Model (II)	Model (IV)
FP: 0 = LA	−0.145 *** (0.041)	−0.275 *** (0.077)	0.220 *** (0.041)	0.459 *** (0.081)
DC				
age	0.004 *** (0.002)	0.008 *** (0.003)		
MS			0.160 *** (0.036)	0.315 *** (0.071)
EDUL			0.014 *** (0.004)	0.027 *** (0.007)
PRB	−0.086 *** (0.030)	−0.165 *** (0.055)		
MI	−0.136 *** (0.043)	−0.245 *** (0.080)		
HL				
SAH	0.592 *** (0.016)	1.101 *** (0.029)	−0.442 *** (0.016)	−0.087 *** (0.031)
DAA	0.034 *** (0.008)	0.064 *** (0.014)	−0.020 *** (0.008)	−0.044 *** (0.015)
FC				
FLS	0.179 *** (0.022)	0.332 *** (0.042)	−0.162 *** (0.023)	−0.321 *** (0.044)
FPEC			0.372 ** (0.181)	0.810 ** (0.354)
LS				
SF			−0.077 ** (0.031)	−0.131 ** (0.060)
smoking	−0.085 ** (0.040)	−0.160 ** (0.076)	0.097 ** (0.041)	0.197 ** (0.080)
drinking	−0.092 ** (0.040)	−0.172 ** (0.074)	0.131 *** (0.040)	0.243 *** (0.078)
exercise	−0.361 *** (0.030)	−0.685 *** (0.056)	0.216 *** (0.030)	0.410 *** (0.059)
_cons		2.839 (0.459)		13.376 (0.483)
Pseudo R2	0.077		0.052	
Ajd.R2		0.283		0.198

Note: ***, **, represent significance levels of 1%, 5%, respectively, and standard error terms are in parentheses.

**Table 6:** 2SLS regression results compared with OLS regression results.

	Model (III)	Model (V)	Model (IV)	Model (VI)
FP: 0 = LA	−0.275 *** (0.077)	−13.426 *** (3.785)	0.459 *** (0.081)	8.056 *** (2.395)
IC: 0 = LA		21.550 * (11.198)		−12.361 * (6.957)
DC				
hukou		−1.400 *** (0.510)		0.900 *** (0.321)
age	0.008 *** (0.003)	0.070 *** (0.019)		−0.035 *** (0.012)
MS		5.409 *** (1.185)	0.315 *** (0.071)	−2.782 *** (0.751)
EDUL			0.027 *** (0.007)	
PRB	−0.165 *** (0.055)	−0.756 *** (0.220)		0.381 *** (0.139)
MI	−0.245 *** (0.080)	−0.898 ** (0.376)		0.443 * (0.234)
HL				
SAH	1.101 *** (0.029)	1.069 *** (0.107)	−0.087 *** (0.031)	−0.851 *** (0.067)
DAA	0.064 *** (0.014)		−0.044 *** (0.015)	
FC				
FLS	0.332 *** (0.042)		−0.321 *** (0.044)	
FPEC		11.593 *** (3.968)	0.810 ** (0.354)	−5.934 ** (2.394)
LS				
SF			−0.131 ** (0.060)	
smoking	−0.160 ** (0.076)		0.197 ** (0.080)	
drinking	−0.172 ** (0.074)		0.243 *** (0.078)	0.3628 * (0.159)
exercise	−0.685 *** (0.056)	−0.950 *** (0.224)	0.410 *** (0.059)	0.564 *** (0.141)
_cons	2.839 *** (0.459)	−5.066 (4.474)	13.376 (0.483)	17.960 *** (2.720)

Note: ***, **, and * represent significance levels of 1%, 5%, and 10%, respectively, and standard error terms are in parentheses.

## Data Availability

The data presented in this study are available on request from the corresponding author. The data are not publicly available due to confidentiality.
